# Treatment of proximal patellar tendon rupture with custom-made anchor-like plate and suture: cases report and literature review

**DOI:** 10.3389/fsurg.2023.1170760

**Published:** 2023-05-09

**Authors:** Hongfei Qi, Zhong Li, Teng Ma, Cheng Ren, Yibo Xu, Qiang Huang, Kun Zhang, Ming Li

**Affiliations:** ^1^ Department of Orthopaedics and Trauma, Hong Hui Hospital, Xi'an Jiaotong University College of Medicine, Xi'an, China

**Keywords:** patellar tendon, rupture repair, bone plates, surgery, case report

## Abstract

We reported 2 cases of patellar tendon rupture at the lower pole of the patella. For patellar tendon rupture, simple suture fixation has been proved to be inadequate in strength. Our center uses custom-made anchor-like plate and suture to treat proximal patellar fracture. The fixation strength is reliable, no additional bone tunnel is required, and the fixation of the lower patellar fracture can be achieved at the same time. After the operation, the patient starts functional exercise of the knee joint at an early stage, The function of the knee joint of the patient recovered well after 1 year, without other complications.

## Introduction

Rupture of patellar tendon is a rare injury, and the incidence rate reported in the literature is (0.68/100,000 people) (1). The rupture of patellar tendon is usually caused by indirect violence (1). Under normal circumstances, the patellar tendon in the knee joint extension position is the most relaxed. With the increase of knee flexion angle, its traction force will also increase accordingly. The rupture of patellar tendon often occurs in this state. In the case of knee flexion, the quadriceps femoris muscle contracts strongly, and the patellar tendon breaks when it is passively stretched beyond its load. The length of patellar tendon is about 3.5–5.5 cm (2). It is very difficult for patellar tendon to break under physiological conditions. Metabolic disorder, systemic diseases such as obesity, chronic kidney disease, gout, diabetes, hyperparathyroidism, abuse of hormones, long-term minor injury and local blocking injection that cause degeneration of patellar tendon are very important factors for its breaking (3, 4).

The main symptom of acute patellar tendon rupture is extension lag. For the typical patellar tendon rupture caused by the strong contraction of quadriceps femoris, it is often accompanied by severe pain. When injured, the specific radiological sign is the higher patella (5). Once the patellar tendon rupture is diagnosed, it should be operated to restore its continuity (6). Due to the abundant blood supply of the patellar tendon, it is generally easy to heal after the repair of acute injury (7). Simple suture of patellar tendon has been proved to lack sufficient strength, and the fixation should be strengthened to avoid secondary fracture (8). Common enhancement repair methods include cerclage wires, Dall-Miles cables, nonabsorbable defects, autologous hamming grafts or tendon allografts (9–11). However, there are complications of skin necrosis, infection and material rupture after cerclage wires and Dall-Miles cables (11, 12). Allograft has problems of disease transmission and graft availability. Autogenous tendon transplantation has additional injury at the donor site, and in elderly patients, the strength and quality of tissue may not be reliable (13). The stress of patellar tendon is greater at the connect of bone and tendon, so the fracture at the two poles is more common than that at the middle (14), which is more common at the lower pole of patella. Two patients with proximal patellar tendon rupture were treated in our center, and the custom-made anchor-like plate combined with suture method was used for surgical treatment, which achieved good clinical results. The report is as follows.

## Case presentation

**Figure 1:**
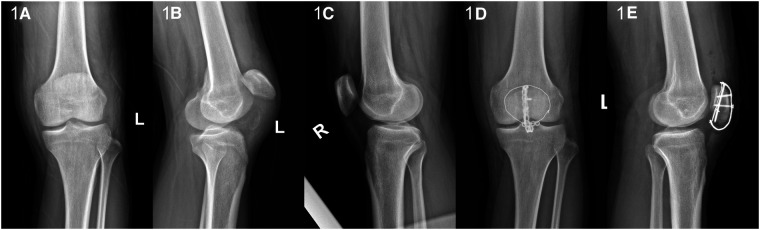
(**A,B**) The anterior and lateral x-ray film of knee joint of affected limb; (**C**) The x-ray film of healthy knee joint before operation; (**D,E**) the postoperative anterolateral x-ray film of knee joint.

Case 1: A 37-year-old male patient suffered from left knee pain and activity limitation for 1 day due to falling on the stairs. The patient's daily activity is not high, with the BMI (Body Mass Index) is 31.2. Complete x-ray and CT (Computed Tomography) examinations of the left knee joint after admission (Figures 1A–E). The Insall-Salvati ratio was 64.71/48.03 (1.35) and the diagnosis was “1. Rupture of the left patellar tendon 2. Avulsion fracture of the starting point of the left patellar tendon at the lower pole of the left patella.” Complete preoperative preparation and selective surgical treatment.

**Figure 2:**
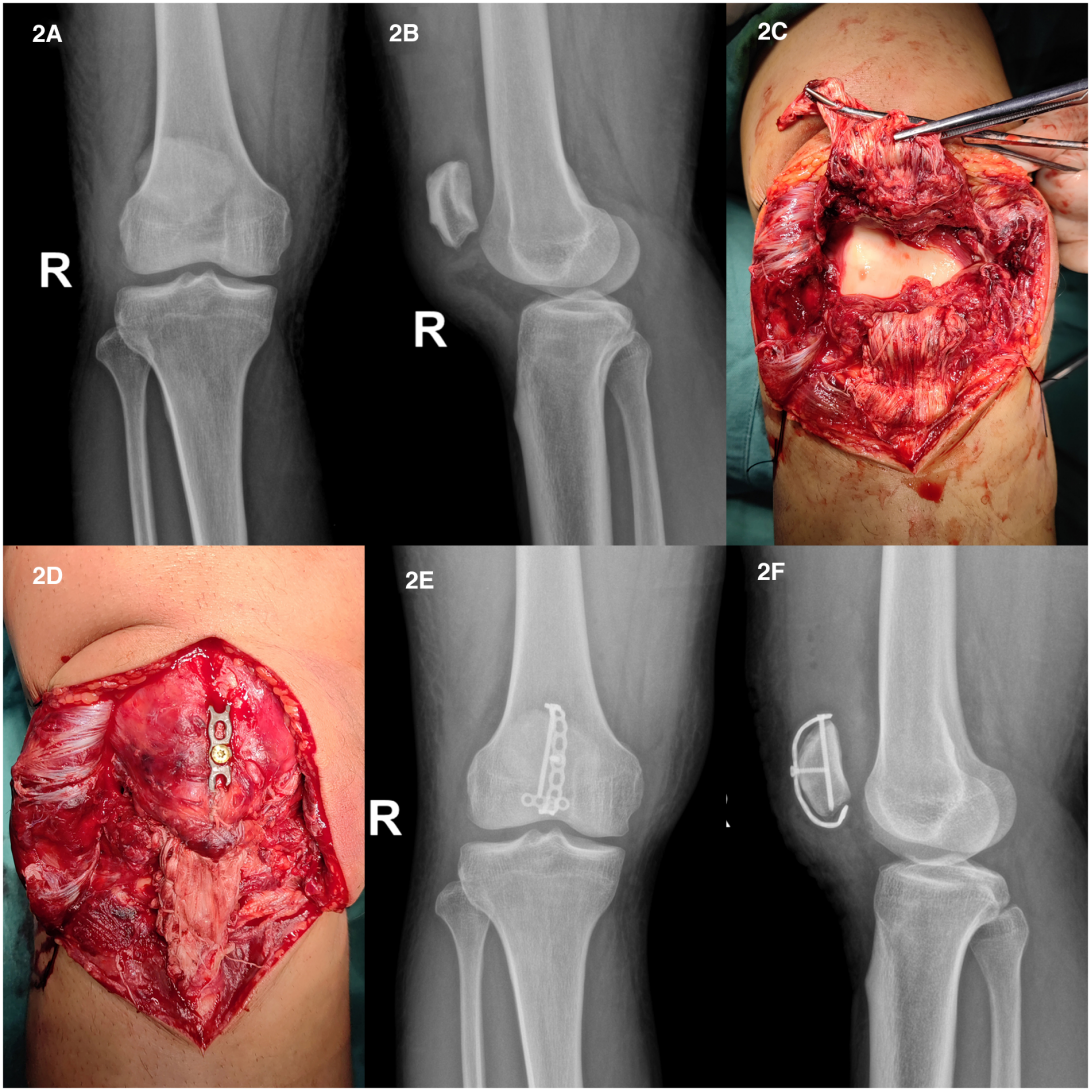
(**A,B**) The anterior and lateral x-ray film of knee joint; (**C**) complete rupture of patellar tendon; (**D**) after the continuity of patellar tendon was reconstructed, the fixation was completed; (**E,F**) the anterior and lateral x-ray films of knee joint after operation.

Case 2: A 34-year-old male patient suffered from pain in the right knee and activity limitation for 1 day due to sprain during walking. The patient's daily activity is not high, with the BMI is 26.7. Complete relevant inspection after admission. The Insall-Salvati ratio was 52.23/42.45 (1.23) and the diagnosis was “rupture of the right patellar tendon.” Active preoperative preparation was made (Figures 2A–D), and the surgical treatment was selected after eliminating the contraindications.

## Surgical technique

**Figure 3:**
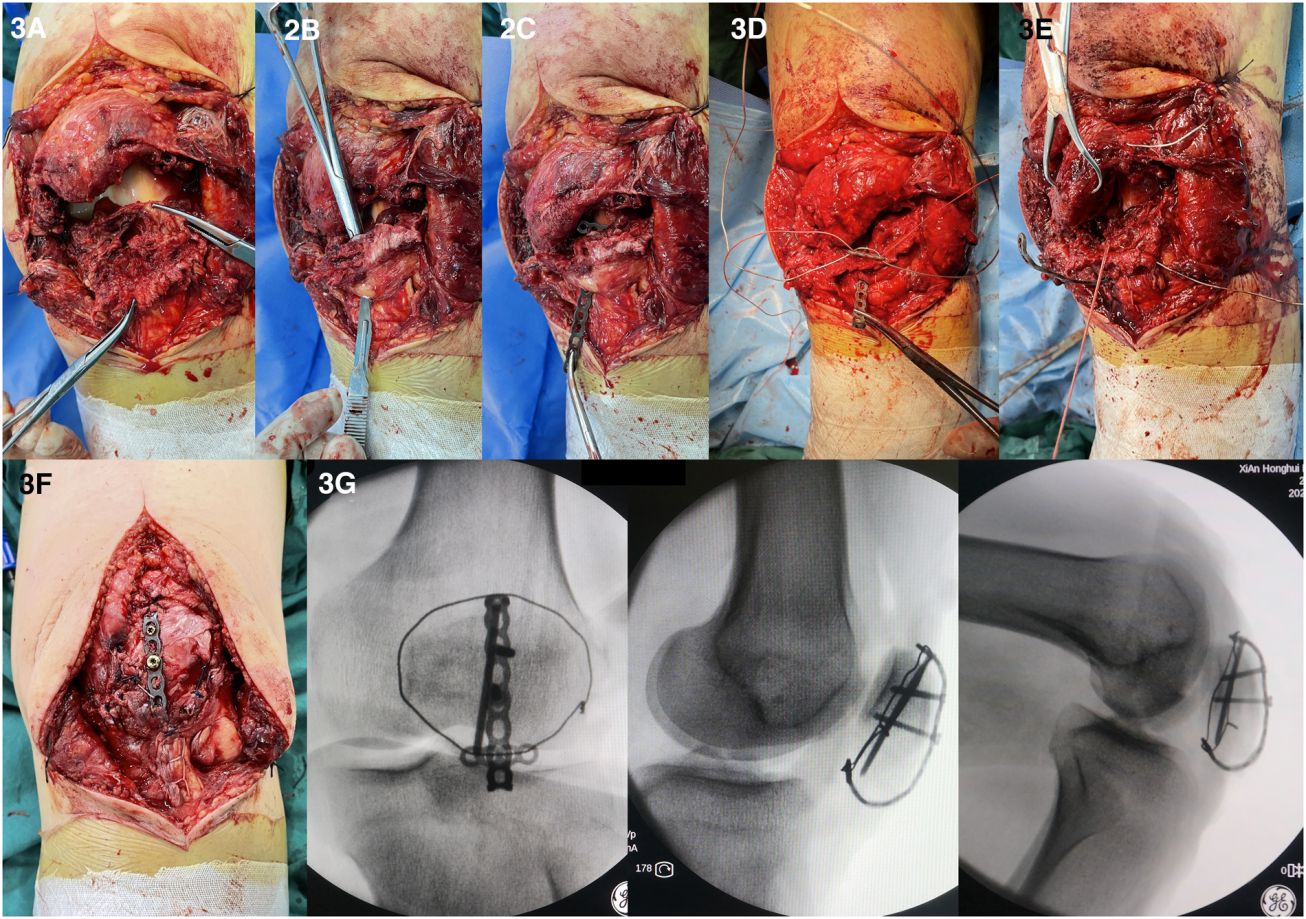
(**A**) The patellar tendon is broken from the lower pole of the patella, and the lower pole is avulsion fracture; (**B**) make a small opening in the distal patellar tendon; (**C**) the T-shaped plate reversely passes through the orifice, and the transverse arm is hung on the patellar tendon; (**D**) the steel wire and suture pass through the transverse arm hole of the plate for use; (**E**) the suture “Z” weaves the distal patellar tendon and passes through the transverse arm hole of the plate for use; (**F**) fix the plate with screws after tightening the steel wire and knotting the suture; (**G**) the x-ray fluoroscopy showed good fixation and internal fixation.

After satisfactory anesthesia, the patient took supine position, the left lower limb was routinely disinfected, and the sterile operation sheet was laid, and the upper leg root was covered with the air bag tourniquet. Take the left knee anterior median incision through the surgical approach, cut the skin, subcutaneous tissue and deep fascia layer by layer, peel and expose the starting point of the patellar tendon at the lower pole of the patella, which shows a cap-like avulsion fracture, the patella moves up and the tension of patellar tendon disappears (Figure 3A). Carefully clean up the blood stasis and clots between the joints and fracture ends, wash the joint cavity, take the T-shaped miniature plate (produced by Tianjin Zhengtian Company, China) and shape it, then penetrate the steel wire to form the “anchor loop device”, insert the sharp knife into the plate reversely at the opening of the patellar tendon (Figures 3B–D), hang the transverse arm of the plate on the patellar tendon and the lower pole of the patella, and pull the patellar tendon towards the proximal end to reduce the fracture, and see that the tension of the patellar tendon is restored, and the x-ray fluoroscopy machine shows that the plate has a good fit. Take the anchor nail suture, one suture is woven and sewn on both sides of the patellar tendon through the hole position of the plate transverse arm, and tighten the knot and fix it on the plate transverse arm; Take another suture and suture it in the “Z” shape in front of the patellar tendon and then pass through the transverse arm of the plate for standby (Figure 3E). Then pull the steel wire from both sides through the internal and external support belt and tighten it, tighten and fix the front two sutures on the plate transverse arm, and finally fix the bone plate with three screws (Figure 3F). It can be seen that the fixation is satisfactory, the intraoperative activity is good, the fracture fixation and patellar tendon tension are restored, and the x-ray fluoroscopy machine shows that the fracture reduction is satisfactory, and the internal fixation positioning is good (Figure 3G). A large amount of salt water was used to wash the wound repeatedly, and a drainage tube was placed in the wound. After the bleeding was strictly stopped, the skin margin was trimmed, and the patellar support band torn on both sides was strengthened. After that, the wound was closed layer by layer, and the sterile dressing was pressurized and bandaged, and the air bag tourniquet was loosened.

## Post-operation procedures

The drainage tube was removed on the second day after operation, and the passive rehabilitation exercise was started on the second day, and the range of activities without flexion limitation was carried out. Once the patient can bear the pain, the active movement of the knee joint is allowed without restriction, especially the extension of the knee joint. The patient began to perform weight bearing walking around 12 weeks after surgery. Professional surgeons will provide rehabilitation guidance and follow-up to patients 1 month, 3 months, 6 months and 12 months after operation. The flexion and extension range of the patient's knee joint was measured one year after the operation and the knee joint function was evaluated by the Lysholm knee scoring scale (15).

## Results

The two patients were followed up for at least 12 months. The avulsion fracture of the lower patella healed smoothly without any complications such as poor wound healing, infection and secondary surgery. One year after the operation, the knee function of the patient returned to normal, without limitation of knee flexion or extension, and without knee pain. The range of motion of knee joint in 2 patients was 5° extension—140° flexion (case 1) and 5° extension—135° flexion (case 2). The Lysholm knee scoring scale in 2 patients was 90 points (case 1) and 95 points (case 2). More cases details can be viewed in the supplementary materials.

## Discussion

The patellar tendon starts from the lower pole of the patella and ends at the tibial tubercle. There is a layer of aponeurosis around it, with rich blood supply. After the rupture of the patellar tendon, the local swelling, the normal contour of the patellar tendon disappeared, the depression could be touched, the active knee extension was weak, the knee joint could not be straightened, and the leg could not be straightened (16). The two cases in this study were caused by pure mechanical violence, and the patients themselves did not have any other diseases. x-ray examination of some patients can find that they are combined with fracture of the lower pole of the patella or avulsion of the tibial tubercle. For patients with complete rupture of the patellar tendon, changes in the upper and lower positions of the patella can occur. Bilateral x-ray films can be further compared. Ultrasound images can clearly reflect the abnormalities of the surrounding tissues of the patellar tendon contour, and have high value in the diagnosis of patellar tendon rupture and early follow-up after surgery, and the accuracy of diagnosis can reach 100% (3, 17). Magnetic resonance examination is of great value in the diagnosis of complete or incomplete rupture (18). In addition, magnetic resonance has unique advantages in the localization of injury, which is conducive to the formulation of preoperative plans and the selection of surgical technology (19). The purpose of repairing patellar tendon rupture is to restore the knee extension function of the knee joint. Surgical repair is the gold standard for patellar fracture. However, there is no perfect surgical technique to solve this problem (3).

The rupture of patellar tendon can be divided into three types according to the location of the rupture: the rupture at the lower pole of the patella, the rupture and avulsion at the tibial tubercle, and the rupture at the middle part of the patellar tendon. Some studies have shown that patellar tendon rupture mostly occurs at the connect of tendon and bone (20). For patients with patellar tendon rupture from the lower pole of the patella, the traditional surgical method requires a transverse slot in the horizontal plane of the lower pole of the patella to accommodate the broken end of the patellar tendon, and then three tunnels are drilled longitudinally from the lower pole of the patella to the upper pole. After the patellar tendon is longitudinally braided and sutured, the suture is pulled out from the lower pole of the patella through the tunnel to the upper pole, and the knot is tightened (21). When the position of the tunnel is deviated, it is easy to damage the articular cartilage. Multiple longitudinal drilling will unnecessarily damage the quadriceps tendon. Finally, the broken end of the patellar tendon is pulled through the suture of the bone tunnel to enter the bone tunnel, making the patellar tendon that has been cleared shorter, resulting in the low patella after the operation.

Many other surgical methods reported in the literature, including wire ligation, allograft tendon transplantation, autologous hamstring tendon transplantation and artificial synthetic ligament enhanced suture (13, 21, 22). Allograft has problems of disease transmission and graft availability (22). Autogenous tendon transplantation has additional injury at the donor site, and in elderly patients, the strength and quality of the transplanted tendon may not be reliable. Maxime Core et al. reported a study using artificial ligament to enhance patellar tendon repair has achieved good clinical results. However, the high cost (a synthetic ligament costs € 476.85, our plates costs is currently approximately € 130) (13), may lead to its failure to popularize widely. In this study, we used custom-made anchor-like plate and suture to treat 2 patients with patellar tendon rupture at the lower pole of the patella, and achieved good clinical results. The following report is presented.

The patellar position may move after the patellar tendon rupture. In this study, the patella will return to its normal position after the patellar ligament is reconstructed with custom-made anchor-like plate and suture technology. One year after operation, the patient recovered normal range of motion, function of knee joint and did not show pain of knee joint. Previous studies have shown that osteolysis of the tunnel will occur on the x-ray after the patellar tendon is fixed through the bone tunnel, which may mean that the reliability of the fixation is weakened. The same situation also occurred in the study of Roudet et al. (23). Our fixation technology does not need to build additional bone tunnels to avoid tunnel osteolysis, because our suture is fixed on the transverse arm of the plate, which provides a stable stop point, and will not occur with bone cutting and internal fixation failure. The transverse arm of the plate drags the proximal end of the patellar tendon to enhance the strength of the patellar tendon fixation and meet the needs of patients for early functional exercise of the knee joint. In addition, this technology can be used to fix the fracture of the lower pole of the patella at the same time. The fracture fragments of the lower pole of the patella in this kind of injury is often very small, and the conventional fixation method may not meet the strength of the fixation (24). This technology can not only reconstruct the continuity of the patellar tendon, but also effectively fix the fracture of the lower pole of the patella. Compared with artificial ligament reinforcement repair, its cost advantage is also obvious.

The key of this technology is the plasticity of the plate, which needs to be well fitted to the surface of the patella. With the continuous proficiency of the operation, this process will become easier and easier. In the selection of screws, we should choose the single cortical screw for fixation. The double cortical screw will damage the articular cartilage. The transverse arm of the plate provides a reliable fixation point for the suture of the patellar tendon, and transfers part of the tension of the patellar tendon to the plate; The transverse arm of the plate can also play a certain protective role in reconstructing the continuity of the patellar tendon and enhance the strength of fixation.

## Conclusion

Patellar tendon rupture is a rare injury, and its main clinical manifestations are pain and walking disorder. Once the patellar tendon rupture is diagnosed, it should be operated to restore its continuity as soon as possible to avoid old injuries or residual knee joint dysfunction. Our center has achieved good clinical results by using custom-made anchor-like plate combined with suture to treat the fracture of the patellar ligament at the lower pole of the patella. This technology enhances the strength of the patellar tendon fixation and does not require additional bone tunnel. In addition, it can also achieve the fixation of the fracture of the lower pole of the patella. This technique has certain advantages for the patellar tendon rupture injury at the lower pole of the patella.

## Data Availability

The raw data supporting the conclusions of this article will be made available by the authors, without undue reservation.
