# Supplementary figures and images for: Treatment of proximal patellar tendon rupture with custom-made anchor-like plate and suture: cases report and literature review
Source: Front Surg. 2023 May 9;10:1170760. doi: 10.3389/fsurg.2023.1170760 (PMC10203210; doi:10.3389/fsurg.2023.1170760)

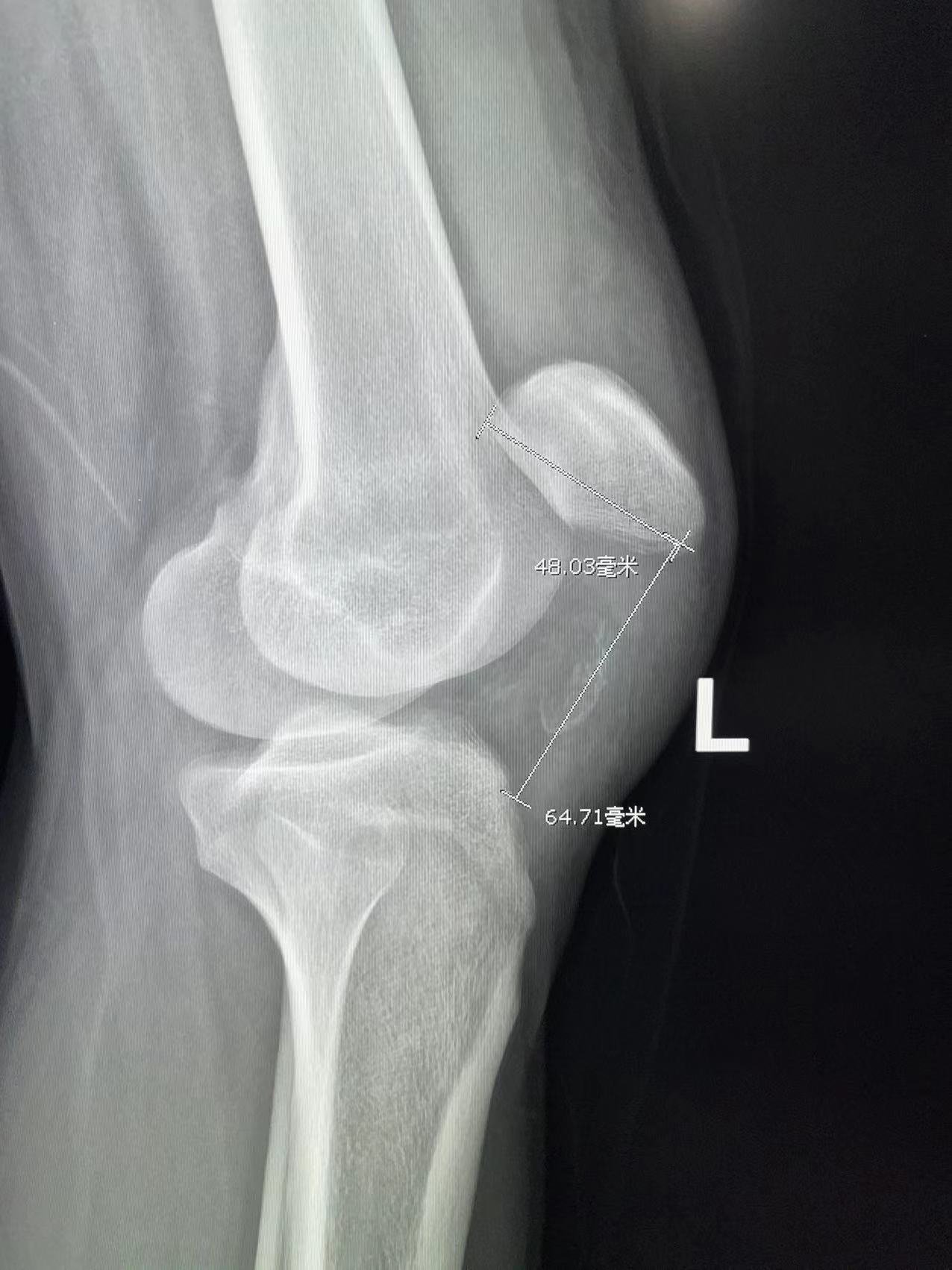

Supplement: Supplementary file 1 [file Image1.jpeg]

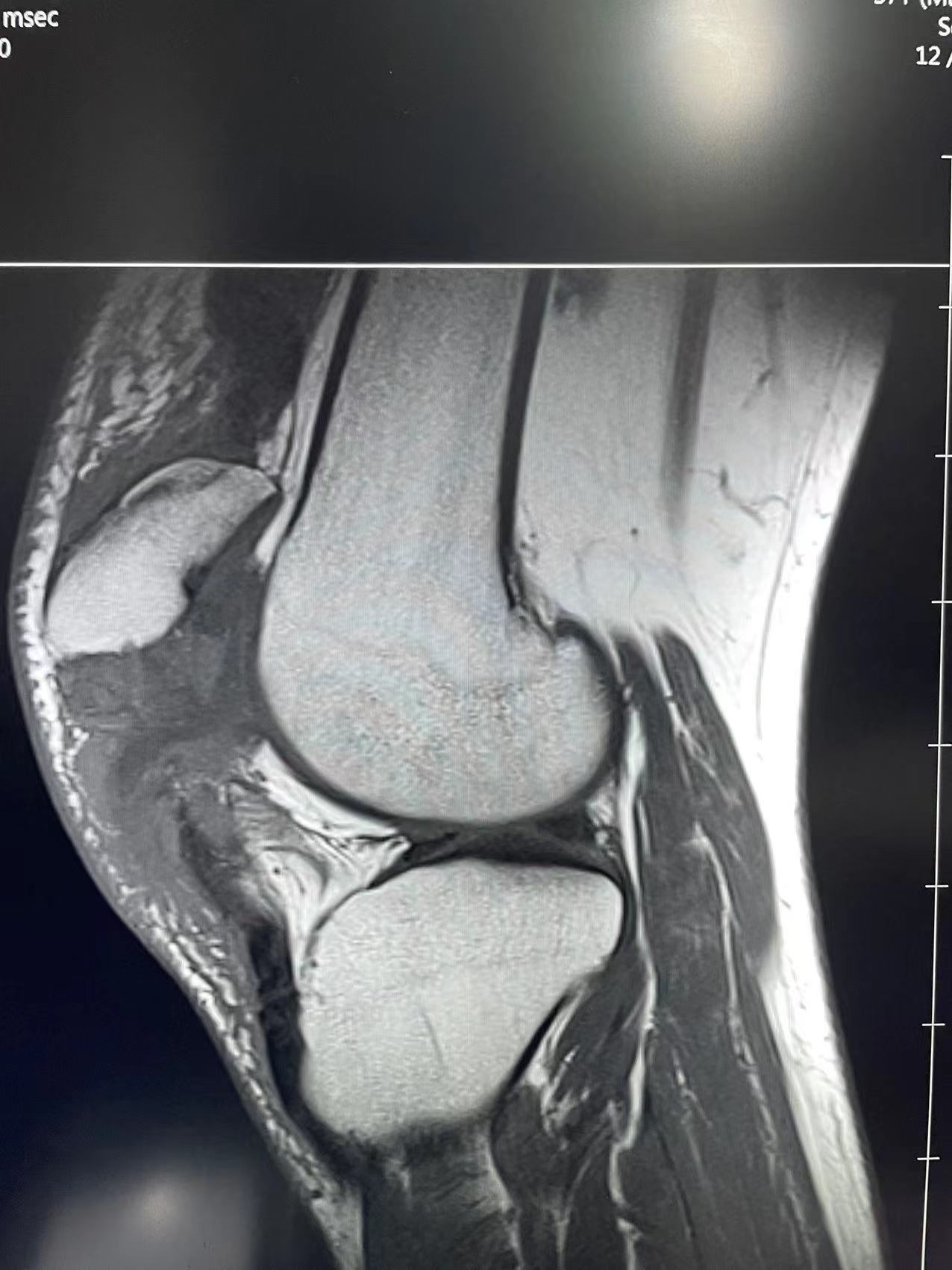

Supplement: Supplementary file 2 [file Image2.jpeg]

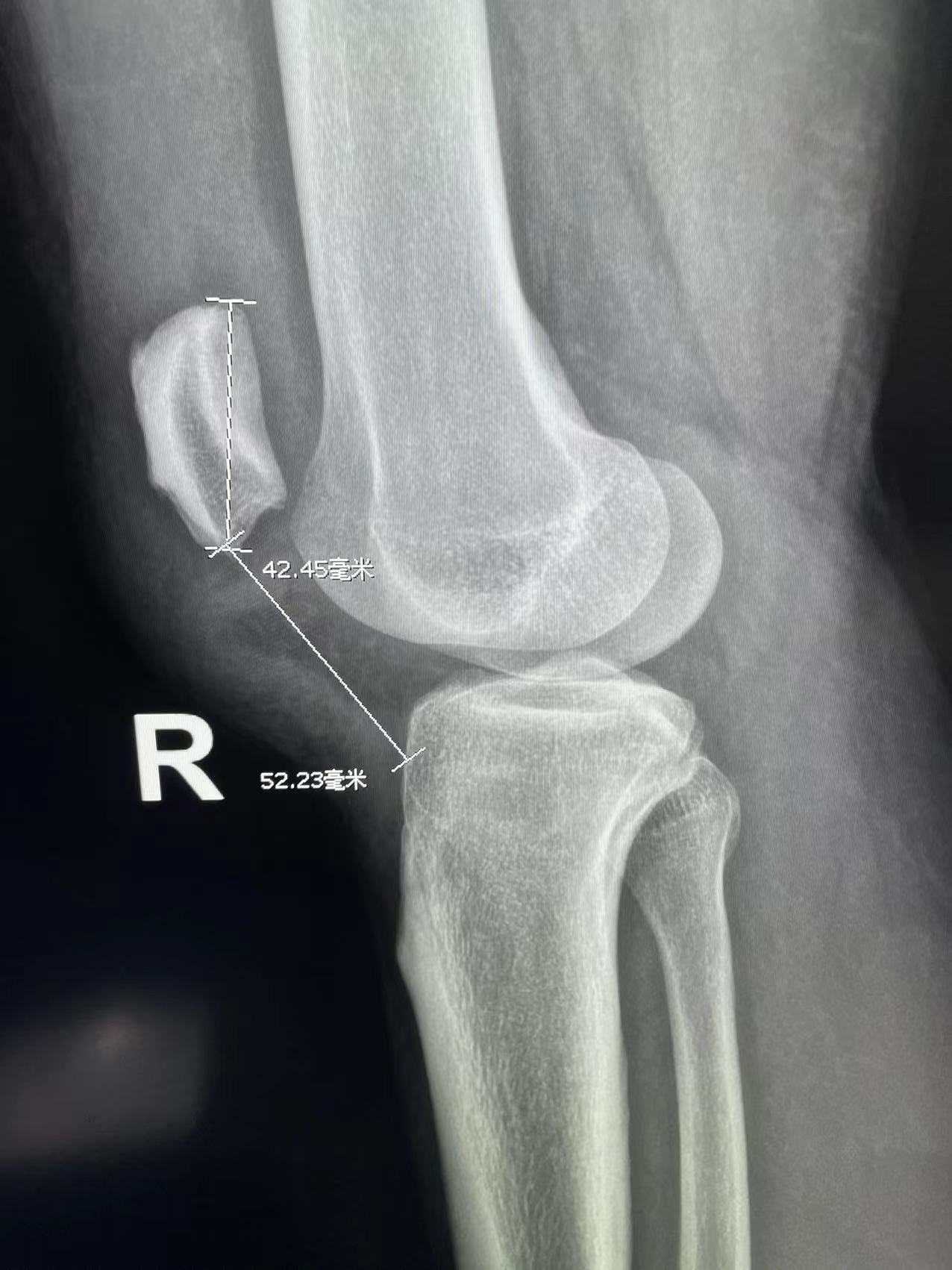

Supplement: Supplementary file 3 [file Image3.jpeg]

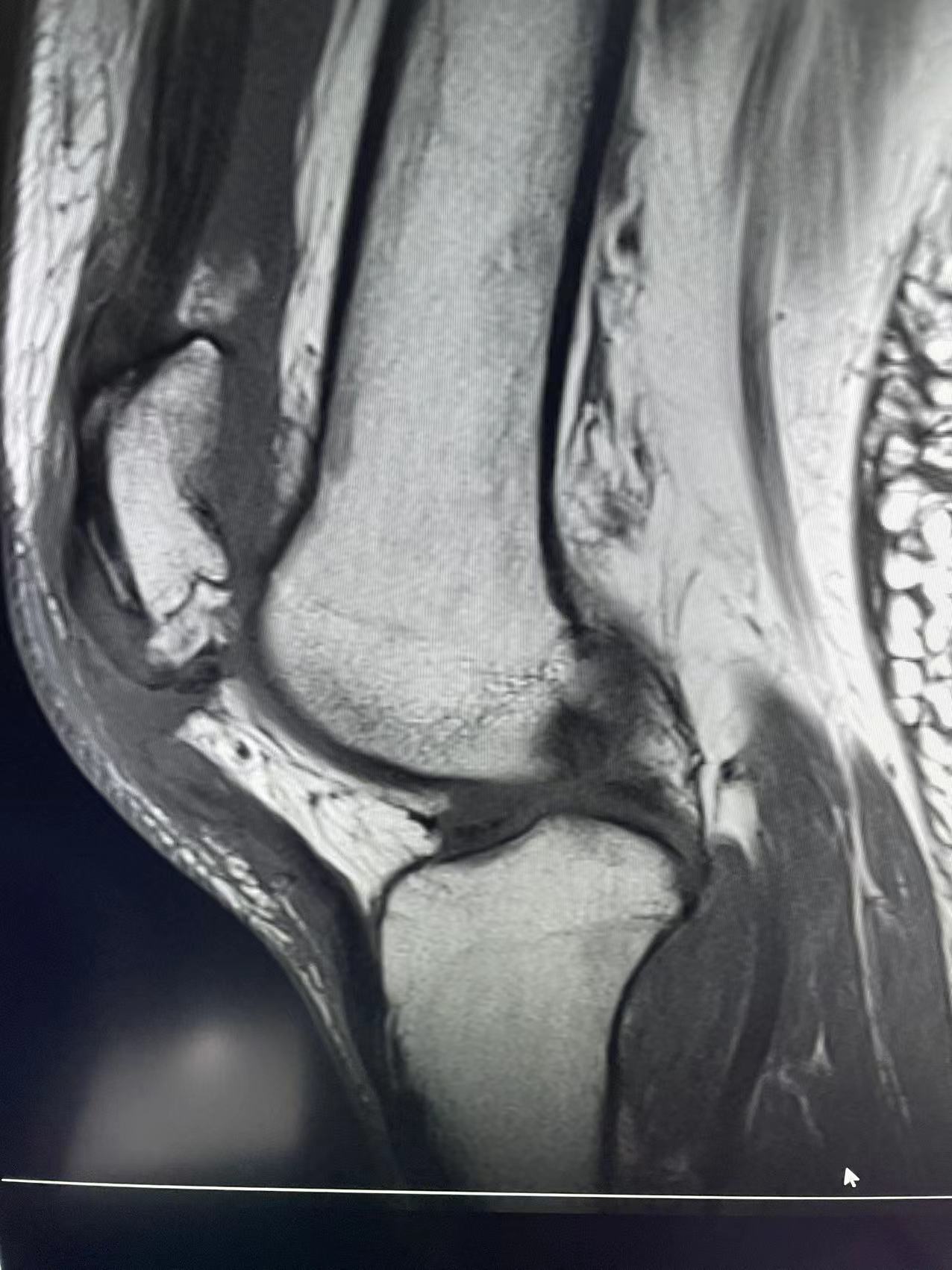

Supplement: Supplementary file 4 [file Image4.jpeg]
